# Chemlistem: chemical named entity recognition using recurrent neural networks

**DOI:** 10.1186/s13321-018-0313-8

**Published:** 2018-12-06

**Authors:** Peter Corbett, John Boyle

**Affiliations:** Data Science Group, Technology Department, The Royal Society of Chemistry, Cambridge, UK

**Keywords:** Chemicals, Named entity recognition, Deep learning

## Abstract

Chemical named entity recognition (NER) has traditionally been dominated by conditional random fields (CRF)-based approaches but given the success of the artificial neural network techniques known as “deep learning” we decided to examine them as an alternative to CRFs. We present here several chemical named entity recognition systems. The first system translates the traditional CRF-based idioms into a deep learning framework, using rich per-token features and neural word embeddings, and producing a sequence of tags using bidirectional long short term memory (LSTM) networks—a type of recurrent neural net. The second system eschews the rich feature set—and even tokenisation—in favour of character labelling using neural character embeddings and multiple LSTM layers. The third system is an ensemble that combines the results of the first two systems. Our original BioCreative V.5 competition entry was placed in the top group with the highest F scores, and subsequent using transfer learning have achieved a final F score of 90.33% on the test data (precision 91.47%, recall 89.21%).

## Introduction

At the Royal Society of Chemistry the data science group undertakes a variety of text mining tasks to enrich both our data offerings and our corpus. One common task is chemical named entity recognition, and the group has spent considerable time applying different machine learning algorithms to extract such information. This paper discusses one of these approaches, which uses structured deep learning.

The chemical entity mention in patents (CEMP) task of BioCreative V.5 [1–3] addresses recognition of chemical named entities in patent text, using a training set of 21,000 patent abstracts and a test set of 9000 patent abstracts. In the previous BioCreative V [4] competition the corresponding named entity recognition task was dominated by systems employing conditional random fields (CRF)—with only two rule-based non-CRF machine learning approaches being used to address the sequence labelling problem. CRF-based systems, such as the highly successful tmChem system [5], treat a sentence or paragraph as a sequence of tokens, and assign a tag to each token to indicate whether it is part of and its position in a chemical name.

A number of popular tagging schemes for named entity recognition (NER) exist. These include: BIO tags, indicating whether a token is at the Beginning, Inside or Outside a named entity; and SOBIE, which has additional tags to BIO tagging for the End of a named entity, and Single token named entities. These systems first assign features to the tokens—representation of what the suffix of the token is, what character n-grams it contains, whether it appears in various dictionaries, etc.—and often features to represent features of neighbouring tokens, or combinations of features across multiple tokens. Having assigned features to tokens, the system then attempts to find the most likely tag sequence given the token features, taking into account both the probabilities of observing a tag given a set of features, and the probabilities of observing a tag given neighbouring tags.

The recent resurgence of artificial neural network techniques known as “deep learning” [6] suggest that these may provide an alternative or a complement to the ubiquitous CRFs. Recurrent neural networks offer an approach to sequence labelling, a common approach to natural language processing (NLP) tasks such as part-of-speech (POS) tagging and named entity recognition. One type of network—a variety of long short-term memory (LSTM) known as a bidirectional LSTM has achieved state-of-the-art performance on common natural language processing (NLP) tasks [7]. In this paper we demonstrate how Bidirectional LSTMs, implemented using the Keras toolkit [8], can be applied to chemical named entity recognition.

The neural network approach has numerous potential advantages. One potential advantage is that recurrent network can carry rich information from token to token (and not just a simple tag transition probability), potentially removing the need for features that look at neighbouring tokens. A second advantage is that deep network allows systems to learn good intermediate representations of tokens, potentially reducing the need for feature engineering. Finally, neural networks are suited to transfer learning, where network components are trained on some task related to the main task which can result in them doing better than those that are randomly initialised. All of these advantages could allow LSTM-based systems to improve upon traditional CRF-based systems.

In this paper we discuss three different approaches to LSTM-based chemical named entity recognition. The first LSTM approach (the “traditional approach”) works similarly to CRF approaches, the second (“minimalist approach”) uses sequences of characters rather than words, and the third approach is an ensemble of both the traditional and minimalist systems. These approaches were used to produce entries for the BioCreative V.5 challenge. We present these systems here, with minor modifications made to ensure that the system can be distributed in a form that produces repeatable results, does not depend upon proprietary datasets, and can make full use of graphical processing unit (GPU) acceleration for fast performance. The original unmodified implementation is presented in Ref. [9]. After the BioCreative V.5 challenge, we improved the system further by making use of transfer learning.

The first system—the “traditional” system—works similarly to traditional CRF-based systems, in that it assigns tags to a sequence of tokens, each token bearing features from a rich feature set. Our “traditional” system differs from those that are CRF-based in a number of ways—for example, our traditional system supplements the feature set with neural word embeddings, and does not include information about neighbouring tokens in the feature set, instead relying on the neural network structure to carry the information from neighbouring tokens to the right place.

The second system—the “minimalist” system—labels a sequence of characters, rather than words (i.e. it does not use a tokeniser), and does not use a rich feature set, instead using character embeddings and multiple LSTM layers in order to induce the equivalent of a feature set internally. In related work, character embeddings have been used in domains where word segmentation is difficult, for example Chinese NLP [10] and text containing programming language snippets [11]—suggesting that this may be particularly suitable for chemical text, where tokenization presents particular difficulties.

Finally, the ensemble system combines the outputs from both the traditional and minimalist system to examine to what extent the two approaches are complementary.

After the competition, we augmented our models using transfer learning. Transfer learning is where a machine learning system is trained on one task, and then parts of the trained system are incorporated into a new network which is then trained on a different task, with the aim of transferring some of the knowledge gained in the first task to the second task. In NLP systems, this can be done by training on “language modelling” tasks—i.e. predicting the probability of observing some token given a context for that token. There is also a variation, “negative sampling”, which looks at a context for a token, and either takes the token from that context (a “positive sample”) or randomly samples one (a “negative sample”), and trains a system to distinguish negative samples from positive.

A common application of transfer learning is the use of neural word embeddings. An embedding layer maps from tokens to *n*-dimensional vectors (often n = 300), and can be trained as part of a larger neural network. Often initial training is done using a negative sampling task. This was pioneered by Collobert et al. [12] as part of their SENNA (semantic/syntactic extraction using a neural network architecture) system. Later improvements were made by Mikolov et al. [13] in their word2vec system, and by Pennington et al. [14] in the GloVe (global vectors for word representation) system. The GloVe system is useful, in that it provides both embedding vectors trained on corpora including Wikipedia and the Gigaword corpus, and the software for users to train their own.

Transfer learning can also be used beyond a single embedding layer. Collobert et al. were able to show transfer between part of speech (POS) tagging, chunking, named entity recognition and semantic role labelling tasks in their SENNA system. Recently, we showed that a negative sampling transfer learning system could be used to improve performance in a chemical-protein interaction detection system [15].

Another approach to transfer learning is to learn a character-level language model. Radford et al. [16] trained a byte-level language model on product reviews, and were able to use this model to train a sentiment analysis system with high data efficiency. One advantage of character-level models is that the number of possible characters in any given context is quite small, making it possible to generate probabilities for all possible characters and thus avoiding the need for negative sampling.

Based on these successes we decided to apply character-level transfer learning to our minimalist system and custom embeddings to our traditional system.

## Methods

ChemListem makes use of two NER systems that can be used independently, or as part of an ensemble.

The first system—the “traditional” system—works similarly to traditional CRF-based systems, in that it assigns tags to a sequence of tokens, each token bearing features from a rich feature set. Our “traditional” system differs from those that are CRF-based in a number of ways—for example, our traditional system supplements the feature set with neural word embeddings, and does not include information about neighbouring tokens in the feature set, instead relying on the neural network structure to carry the information from neighbouring tokens to the right place.

The second system—the “minimalist” system—labels a sequence of characters, rather than words (i.e. it does not use a tokeniser), and does not use a rich feature set, instead using character embeddings and multiple LSTM layers in order to induce the equivalent of a feature set internally. In related work, character embeddings have been used in domains where word segmentation is difficult, for example Chinese NLP [10] and text containing programming language snippets [11]—suggesting that this may be particularly suitable for chemical text, where tokenization presents particular difficulties.

Finally, the ensemble system combines the outputs from both the traditional and minimalist system to examine to what extent the two approaches are complementary.

After the competition, we augmented our models using transfer learning. Transfer learning is where a machine learning system is trained on one task, and then parts of the trained system are incorporated into a new network which is then trained on a different task, with the aim of transferring some of the knowledge gained in the first task to the second task. In NLP systems, this can be done by training on “language modelling” tasks—i.e. predicting the probability of observing some token given a context for that token. There is also a variation, “negative sampling”, which looks at a context for a token, and either takes the token from that context (a “positive sample”) or randomly samples one (a “negative sample”), and trains a system to distinguish negative samples from positive.

A common application of transfer learning is the use of neural word embeddings. An embedding layer maps from tokens to *n*–dimensional vectors (often n = 300), and can be trained as part of a larger neural network. Often initial training is done using a negative sampling task. This was pioneered by Collobert et al. [12] as part of their SENNA (semantic/syntactic extraction using a neural network architecture) system. Later improvements were made by Mikolov et al. [13] in their word2vec system, and by Pennington et al. [14] in the GloVe (global vectors for word representation) system. The GloVe system is useful, in that it provides both embedding vectors trained on corpora including Wikipedia and the Gigaword corpus, and the software for users to train their own.

Transfer learning can also be used beyond a single embedding layer. Collobert et al. were able to show transfer between part of speech (POS) tagging, chunking, named entity recognition and semantic role labelling tasks in their SENNA system. Recently, we showed that a negative sampling transfer learning system could be used to improve performance in a chemical-protein interaction detection system [15].

Another approach to transfer learning is to learn a character-level language model. Radford et al. [16] trained a byte-level language model on product reviews, and were able to use this model to train a sentiment analysis system with high data efficiency. One advantage of character-level models is that the number of possible characters in any given context is quite small, making it possible to generate probabilities for all possible characters and thus avoiding the need for negative sampling.

Based on these successes we decided to apply character-level transfer learning to our minimalist system and custom embeddings to our traditional system.

For each of our approaches there was a three step process, involving pre-processing, a neural network step, and finally post-processing. These steps are detailed below, additionally details of extensions we have applied to our original Biocreative entry are also given.

### Pre-processing

Tokenisation in the traditional system was performed using a modified version of a Python translation of the Oscar4 tokeniser [17]. On the training data only, when an entity boundary was in the middle of a token, the token was split in two. The minimalist system does not use tokenization—however individual characters can be treated as tokens. Tokens in the training data were assigned SOBIE (sometimes known as BIOES) tags—”O” marking a token not part of an entity, “S” marking a token that is the whole of an entity (a “singleton”), “B” marking a token at the beginning of an entity, “I” marking one inside an entity, and “E” marking one at the end.

For both systems the data was split 80:20 for training and testing.

The traditional system starts with finding those tokens in the corpus that occur more than two times, and assigning initial embedding vectors based on the publically available GloVe embeddings [14]—tokens not found in GloVe are given initial embedding vectors full of zeros. Tokens that occur two times or less are all given a single “unknown token” vector, again initialized to zeros.

The traditional system uses a “preclassifier” [18] to judge how likely a token is to be chemical—i.e. assigned an S, B, I or E tag as opposed to O. To train this, the preclassifier subsystem first finds tokens only ever tagged O or only ever tagged SBIE, then generates binary features for each of these, then selects the 1000 binary features with highest mutual information with O-only vs SBIE-only, and finally uses those to train a random forest (using scikit-learn [19]) with 100 trees. This “preclassifier” is used for producing scores (probability predictions) for tokens it was not trained on. The system trains an additional 5 preclassifiers each using four fifths of the available tokens, and uses each to produce a score for the remaining one fifth. The features for the preclassifier are: word shape, character 4-, 3-, 2- and 1-grams (including start and end markers, so this gets prefixes and suffixes), tests against various regular expressions, and tests to see if the token is in various lexicons (a list of chemicals derived from ChEBI (chemical entities of biological interest) [20], a list of chemical elements, and a standard English word list).

Additionally, there are two sets of features that are sent directly to the neural network. One set includes length-based measures (including the number of all non-lowercase characters, the number of all non-letter characters and the number of digit characters) as numerical features, and binary features for the lexicons and regular expressions above. This set is passed to the network in its entirety. The second set of features consists of the 100 most common binary features selected from 2- and 3-character suffixes and word shapes. The features for each token in a sentence (excluding the embeddings) consist of the score from the preclassifier and the two sets of features from the paragraph above.

The minimalist system uses only character embeddings—a set of 90 characters (letters, digits, common punctuation) is used, with an “unknown character” character acting as the 91st character.

### Neural network

The traditional network is as shown in Table 1. It has two inputs—input **ti1** is a sequence of integers, one per token, indicating which token is at which point, whereas input **ti2** contains the other features as described above. The output layer **td1**, a time-distributed dense layer, with 5 outputs per token (corresponding to S, O, B, I and E tags), with a softmax activation function—this ensures that the outputs for each token sum to 1.

**Table 1:** The layers used in the traditional network

Layer	Type	Input(s)	No. of output neurons	Notes
**te1**	Embedding	**ti1**	300	
**tc1**	Conv1D	**ti2**	256	Width = 3, activation = relu, dropout of 0.5
**tm1**	Concatenate	**te1, tc1**	556	
**tb1**	Bidirectional LSTM	**tm1**	64 per direction, total 128	Dropout of 0.5
**td1**	TimeDistributed Dense	**tb1**	5	Activation = softmax

The embedding layer **te1** was initialised using a set of embeddings that had been produced by the GloVe project [14]—these 300-dimensional vectors had been trained on Wikipedia 2014 and the Gigaword 5 corpus.

The system was trained for 20 epochs, with the model being saved after each epoch, and evaluated against the remaining 20% of the data. Each epoch was trained in mini-batches, drawn from batches of sentences all the same length. The model from epoch that gave the best F score was selected.

The minimalist network as shown in Table 2 has a single input (**mi1**)—a sequence of integers, one per character in the input (with 91 possible characters). The output layer is **md1**, and works in the same manner as **td1** in the traditional system.

**Table 2:** Layers in minimalist network

Layer	Type	Input(s)	No. of output neurons	Notes
**ml1**	LSTM	**mi1**	128	
**ml2**	LSTM	**mi1**	128	Reversed
**mm1**	Concatenate	**ml1, ml2**		Dropout of 0.5
**mb1**	Bidirectional LSTM	**mm1**	64 per direction, total 128	Dropout of 0.5
**mb2**	Bidirectional LSTM	**mb1**	64 per direction, total 128	Dropout of 0.5
**md1**	TimeDistributed(Dense)	**mb2**	5	Activation = softmax

This system was trained for 30 epochs. As before, the model from the highest-scoring epoch was selected. The same mini-batch training procedure was used, except that for the first four epochs, the system was trained in order of sequence length, with the shortest sequences first.

Both networks were trained with the root mean square propagation (RMSProp) optimizer, using the categorical cross-entropy loss function. The code was migrated onto GPUs, for speed improvements, which has some limitation when using CuDNNLSTM (CUDA (compute unified device architecture) Deep Neural Network LSTM) as it does not allow the use of recurrent dropout. Further tests are detailed below to show how such a migration affects the performance of the systems.

### Post-processing

The neural network assigns five scores to each token or character—one for each of the S, O, B, I and E tags. To convert this to a list of entities, the system scans for possible entities, looking up the value for each tag in each possible entity in each position, taking the minimum value, and, if this is above a threshold, accepting the entity and assigning it that value as a score. The thresholds were 0.5 for both systems.

The ensemble system works by running both systems with a lower threshold, and generating two lists of entities. If an entity appears in only one list, its score is the score from that list, otherwise it is the sum of the scores from the two lists. This score is then divided by 2, and a threshold of 0.475 is applied. This low threshold below 0.5 was chosen to ensure that an entity detected by only one system—e.g. an entity that starts or ends inside a token, and is thus undetectable by the traditional system—can still be detected by the ensemble.

The BioCreative challenge did not allow for overlapping entities to be submitted, therefore checks were done and in the runs where this was a possibility, the lower-scoring entities were discarded.

### Extensions

We have applied further extensions using transfer learning to our original entry to the Biocreative NER competition. We augmented our chemical NER systems after the BioCreative challenge to study improvements that could be gained using transfer learning techniques. Details of these improvements are outlined below, with the findings being detailed in the results and discussion section.

#### Extensions to traditional system

We have improved the traditional system by replacing the publicly-available GloVe embedding file with a custom-compiled version made using the software based on a more relevant corpus. To do this, we prepared a corpus of patent titles and abstracts from United States Patent and Trademark Office (USPTO) patents with cooperative patent classification (CPC) codes A61K31 or A61P, from 2006 and 2016. The corpus file had one title or abstract per line. Each line was tokenised, with one space character between tokens (so “acetone-based” became “acetone-based”). It was used to train a set of 300-dimensional word embeddings (the “custom embeddings”) using the GloVe software [14].

#### Extensions to minimalist system

The minimalist system was improved by the use of two transfer learning systems. The first system was called “predictive transfer”, and the second system was called “dictionary transfer”.

The predictive transfer system, shown in Table 3, uses a corpus file prepared in a similar manner to the one for the traditional system, but without the tokenisation stem. The system reads in one line at a time, creating an input **mi1** as in the main minimalist system. The outputs **md2** and **md3** contain one-high encodings of the character sequence represented by **mi1**, but shifted one character to the left or to the right. The system thus attempts to predict each character in the sequence based on either all of the characters previous to it, or all the characters after it. The training of this transfer network was fully completed prior to training the main network, using each line from the corpus once.

**Table 3:** “Predictive transfer” network

Layer	Type	Input(s)	No. of output neurons	Notes
**ml1**	LSTM	**mi1**	128	
**ml2**	LSTM	**mi1**	128	Reversed
**md2**	TimeDistributed(Dense)	**ml1**	91	Activation = softmax
**md3**	TimeDistributed(Dense)	**ml2**	91	Activation = softmax

A second transfer system—which we call “dictionary transfer”—worked on a list of words, drawn from a list of chemical names found in ChEBI, a list of element names and an English dictionary. The network is shown in Table 4. The output **md4** is a three-dimensional vector saying whether the word appears in the chemical name list, the element name list and/or the dictionary—essentially, it trains the embedding and LSTM layers of the main minimalist network to recognise whether single words are chemical or not. With this system, training was interleaved with training the main network—training was alternated between mini-batches of transfer training and main-system training, until all of the words used in transfer learning had been used.

**Table 4:** “Dictionary transfer” network

Layer	Type	Input(s)	No. of output neurons	Notes
**ml1**	LSTM	**mi1**	128	
**ml2**	LSTM	**mi1**	128	Reversed
**mm1**	Concatenate	**ml1, ml2**		Dropout of 0.5
**mb1**	Bidirectional LSTM	**mm1**	64 per direction, total 128	Dropout of 0.5
**mb2**	Bidirectional LSTM	**mb1**	64 per direction, total 128	Dropout of 0.5
**mp1**	GlobalMaxPooling1D	**mb2**	128	
**md4**	Dense	**mp1**	3	Activation = sigmoid

We also examined the effects of using different combinations of transfer learning schedules—for each transfer learning system, either completing transfer learning before training the main system, interleaving transfer learning with main-system training, or leaving out that training altogether. When both transfer systems were trained before main-system training began, we tried three variations—training the “predictive transfer” system first, training the “dictionary transfer” system first, or interleaving training the two, a mini-batch at a time.

## Results and discussion

The results of the systems evaluated in the BioCreative V.5 event, as described in [9] are shown in Table 5. In the competition our ensemble system entry gave the third best F score, with the judges stating that the top three scores were statistically indistinct [2, 3].

**Table 5:** Results of official BioCreative V.5 submissions

System	Official test			Internal evaluation		
	F (%)	Precision (%)	Recall (%)	F (%)	Precision (%)	Recall (%)
Traditional	89.19	88.67	89.71	87.03	86.48	87.58
Minimalist	89.01	88.65	89.36	86.64	84.79	88.58
Ensemble	*90.32*	*90.02*	*90.62*	*88.07*	*86.46*	*89.76*

The official test was part of the BioCreative competition, and the internal evaluations were performed by ourselves using 1/5 of the training data not used for training

Entries in italics are the best results in that column

As mentioned in the introduction, the systems described here differ slightly from those used to create the BioCreative V.5 entries—there were some changes to remove dependencies on proprietary datasets, allow the use of the GPU, and to ensure that the systems could be distributed as open source. These changes mean that the systems now available give slightly different scores to the originals using BioCreative—these differences are attributed to making speed improvements to the code (e.g. ensuring the code runs on GPUs).

To demonstrate the performance of our systems we present here two evaluations. The first evaluation, called “internal style”, uses our 1/5 of the training data not used for training as in the table above. The second evaluation, called “official style”, replicates the evaluation done during BioCreative V.5, by using those abstracts from the official test set that contained at least one chemical entity in the gold standard annotations. The results are in Table 6.

**Table 6:** Results of systems described in this paper

System	Official test			Internal evaluation		
	F score (%)	Precision (%)	Recall (%)	F score (%)	Precision (%)	Recall (%)
1: Traditional	89.04	89.57	88.52	86.75	86.03	87.49
2: Minimalist	88.71	88.04	*89.38*	86.85	85.10	88.68
3: Ensemble of 1 and 2	90.11	88.69	88.02	88.02	86.89	*89.18*
4: Traditional with custom embeddings	89.19	90.05	87.93	86.91	87.10	86.72
5: Minimalist with transfer training	89.32	90.49	88.18	87.18	87.58	87.38
6: Ensemble of 4 and 5	*90.33*	*91.47*	89.21	*88.17*	*87.99*	88.17

Entries in italics are the best results in that column

As noted above the traditional and minimalist do not perform quite as well as their counterparts used for the original BioCreative entry. The reason for this change in score, we believe, is largely due to the LSTM implementation for the original implementation (which ran on a CPU) using recurrent dropout—a feature not available with the fast GPU-based LSTM implementation later used. The original submission also had used some proprietary datasets, which may have boosted performance.

The custom embeddings on the traditional system do have a positive effect, with a 0.15 to 0.16 percentage point improvement to F score. The transfer learning appears to have boosted the minimalist system by 0.53 to 0.61 percentage points, with the ensemble being improved by 0.15 to 0.22 percentage points.

For completeness we also looked at the impact of using the CuDNNLSTM without recurrent dropout versus using a default LSTM with recurrent dropout. The results are shown in Table 7.

**Table 7:** Results of training using different LSTM implementations

System	Official test			Internal evaluation		
	F score (%)	Precision (%)	Recall (%)	F score (%)	Precision (%)	Recall (%)
1: Traditional	89.04	89.57	88.52	86.75	86.03	87.49
4: Traditional with custom embeddings	89.19	*90.05*	87.93	86.91	*87.10*	86.72
7: As 1, with default LSTM, and recurrent dropout	89.11	89.23	88.98	86.93	85.86	88.01
8: As 4, with default LSTM, and recurrent dropout	*89.26*	89.19	*89.34*	*86.96*	85.86	*88.09*

Entries in italics are the best results in that column

Training system 1 took 23 min for the parts that involved the neural network (and an additional five minutes of preparation) whereas the corresponding parts of system 7 took 163 min—giving a sevenfold speedup. The speedup came at a cost of 0.05–0.18 percentage points of F score.

A breakdown of the transfer learning approaches is shown in Table 8. All scores are for the “internal” test set.

**Table 8:** Results of internal evaluation of minimalist system with different transfer learning strategies

Predictive transfer	Dictionary transfer	F score (%)	Precision (%)	Recall (%)
None	None	86.85	85.10	88.68
None	At start	86.40	85.20	87.64
None	Interleaved	86.80	85.74	87.88
At start	None	87.14	85.47	*88.88*
At start	After predictive	87.08	86.16	88.07
After dictionary	At start	87.24	86.09	88.42
At start	Interleaved with dictionary	87.03	85.46	88.66
At start	Interleaved	*87.38*	*87.18*	87.58
Interleaved	None	87.30	85.90	88.75
Interleaved	At start	86.88	85.59	88.20
Interleaved	Interleaved	87.30	86.36	88.27

Entries in italics are the best results in that column

These scores show that predictive transfer shows a clear advantage—all of the F scores involving predictive transfer are higher than all of the F scores not involving predictive transfer. The benefit of dictionary transfer is less clear—in many cases dictionary transfer worsens performance.

This phenomenon of attempted transfer learning actually reducing performance is known as “negative transfer”. Here, we have mitigated the negative transfer effects from the dictionary transfer by interleaving that transfer learning with training on the main task, and by including predictive transfer learning as well—the best combination uses a block of transfer learning at the start. Two recent reviews of transfer learning [21, 22] have both noted that the area of negative transfer has not been widely researched, and we are not aware of other demonstrations of this interleaving technique being used to prevent negative transfer.

Despite the different methods involved, the traditional and the minimalist system performed similarly. Combining them into an ensemble gives a substantial advantage; about 1 percentage point of F score, giving our best system a final F score of 90.33%, slightly higher than the ensemble submitted to BioCreative V.5. The improvements we have made to the system, and have made available as open source, show a strong increase in training speed, while holding the F score nearly constant.

## Conclusions

We have shown here that using deep learning techniques can give state-of-the-art performance on the chemical named entity recognition problem. Our system scored well in the BioCreative V.5 CEMP evaluation [2], giving the third highest F score—the difference with the two higher-scoring systems [23, 24] was not statistically significant. All three of these systems made use of bidirectional LSTMs, whereas the lower-scoring systems did not—this highlights the importance of LSTM-based methods.

The use of transfer learning has shown to improve the minimalist system by a substantial amount (0.5–0.6 percentage points of F score), with the best transfer learning approach combining multiple transfer learning tasks. The application of similar transfer learning strategies to the traditional system is a possible area for improvement. In [15] we have presented an application of transfer learning in token-based bidirectional LSTMs to the problem of chemical-protein interaction recognition, and are investigating applying the techniques developed there to the named entity recognition problem.

Our best system has achieved an F score of 90.33%—above the symbolic “90% barrier”, which is approaching human-level performance—for example an inter-annotator agreement study of chemical named entity annotation found that an F score of 93% is possible [25]. Further improvements may be possible, and we are investigating ways to do this.

The software used is available as open-source software, at https://bitbucket.org/rscapplications/chemlistem.

## Abbreviations

NER: named entity recognition
CRF: conditional random fields
LSTM: long short term memory
CEMP: chemical entity mentions in patents
NLP: natural language processing
BIO: begin inside outside
SOBIE: single outside begin inside end
GPU: graphical processing unit
POS: part of speech
CPC: cooperative patent classification
CuDNNLSTM: CUDA deep neural network LSTM
CUDA: compute unified device architecture
RMSProp: root mean square propagation
ChEBI: chemical entities of biological interest
SENNA: semantic/syntactic extraction using a neural network architecture
GloVe: global vectors for word representation
